# Foreign Bodies Ingestion in Children: Experience of 61 Cases in a Pediatric Gastroenterology Unit from Romania

**DOI:** 10.1155/2016/1982567

**Published:** 2016-02-01

**Authors:** Smaranda Diaconescu, Nicoleta Gimiga, Ioan Sarbu, Gabriela Stefanescu, Claudia Olaru, Ileana Ioniuc, Iulia Ciongradi, Marin Burlea

**Affiliations:** ^1^“Gr. T. Popa” University of Medicine and Pharmacy, Strada Universitatii, No. 16, 700115 Iasi, Romania; ^2^“St. Mary” Emergency Hospital for Children, Strada Vasile Lupu, No. 62, 700309 Iasi, Romania; ^3^“St. Spiridon” Emergency Hospital, Bulevardul Independentei, No. 1, 700111 Iasi, Romania

## Abstract

The ingestion of foreign bodies is a worldwide pediatric pathology. We assessed the clinical, endoscopic, and therapeutic aspects of this condition in a pediatric gastroenterology unit. We reviewed 61 patients (median age of 3.25 ± 4.7 years). The most frequently ingested objects were coins (26.23%), unidentified metal objects (13.11%), bones (8.19%), batteries, and buttons (6.55%). The clinical features we encountered included abdominal pain (55.73%), vomiting (34.42%), and asymptomatic children (29.5%). Routine X-ray examination enabled finding the foreign body in 42 of the cases. An esophagogastroduodenoscopy was performed within 24–72 hours. 25 cases resulted in a negative endoscopy (40.98%), 19 objects (31.14%) were removed using a polypectomy snare, and extraction failure occurred in 17 patients (27.86%). 28 foreign bodies were passed without incidents; in 14 cases, the swallowed objects were never found. In one case, a battery was stuck in the esophageal folds and led to tracheal-esophageal fistula and bronchopneumonia and later to esophageal stenosis. We report a large proportion of foreign bodies that could not be identified or removed due to lack of early endoscopy and poor technical settings. Batteries and sharp objects lead to severe complications and preschool-age children are at high risk for such events.

## 1. Introduction

Ingestion of foreign bodies is a relatively common problem encountered in pediatric pathology. Children tend to explore the environment by inserting objects in their mouths; some of these items can be inevitably swallowed. Ingestion of foreign bodies is also a significant cause of parental anxiety. The phenomenon of foreign bodies' ingestion is a worldwide problem. The American Association of Poison Control reports around 125,000 ingestions of foreign bodies in people aged 19 years and similar data are available in European countries, as discussed in the specialty literature [1]. The distribution is relatively equal by gender: boys : girls = 1 : 1 and the peak incidence is between the ages of 6 months and 4 years. In teenagers, the ingestion of foreign bodies raises suspicions of psychiatric pathology or risky behaviors, as Klein reported [2]. The objects most frequently swallowed by children are radiopaque ones: coins, screws, batteries, or toy parts [3]. Most complications are caused by impaction of foreign bodies in the esophagus, especially in the case of anatomical defects or preexisting diseases, but the literature also describes cases of appendicitis induced by foreign bodies that were stuck in the cecum [3–5].

## 2. Material and Method

We conducted a descriptive retrospective study over a period of 5 years (2009–2014) in order to assess the particular aspects of foreign body ingestions in children admitted in a pediatric gastroenterology unit from a tertiary care center in Northeastern Romania. All the children with documented cases of foreign body ingestion were included in the study. Exclusion criteria are as follows: documented aspiration of foreign bodies, previous extraction by the otolaryngologists, or elimination prior to admission. The data (age, sex, object type, clinical presentation, endoscopic findings, and therapeutic methods) were collected from patients' files and endoscopic records. As we are a tertiary care center, the patients were referred to us by the emergency room of our hospital where they came on their own or by different county hospitals lacking endoscopy service. All the patients underwent plain thoracic-abdominal X-ray within the first hour of their admission, regardless of the ingestion time and clinical symptoms. Upper digestive endoscopy was performed by the same team of pediatric gastroenterologists after obtaining the respective informed consent. Some of the patients ingested nonradiopaque foreign bodies, so we performed endoscopy even in the case of negative X-rays.

## 3. Results

We encountered 61 cases of foreign body ingestions from a total number of 2,675 upper digestive endoscopies, thus resulting in a frequency of 2.28%. As we are a tertiary care center, the patients were referred to us by the emergency room of our hospital where they came on their own or by different county hospitals lacking endoscopy service. The children included in the study were aged between 10 months and 17 years, with a median age of 3.25 ± 4.7 yrs. Gender distribution included 32 boys (52.45%) and 29 girls (47.54%). Of these, 25 children (40.98%) were in different types of institutionalized settings (kindergarten, centers for children with disabilities, child psychiatry services, and juvenile detention centers), hence not under parental surveillance. The clinical presentation (in various associations) included vomiting (34.42%), abdominal pain (55.73%), foreign body sensation (11.47%), hematemesis (3.27%), drooling and food refusal (1.64%), and stridor and cough (1.64%); 18 patients were completely asymptomatic (29.50%) (Table 1).

We performed a multiple regression analysis in order to establish some correlations between clinical symptoms and different potentially predictive factors (Table 2).

Thirty-eight children (62.35% of patients) presented to the hospital within the first 24 hours after ingestion, 16 children (26.33%) presented within 24–48 hours after ingestion, and only 7 (11.32% of cases) came to hospital later than 72 hours after ingestion. According to the statements of parents and older children, the foreign objects that were swallowed included coins (26.23%), other metal objects (13.11%), bones (8.19%), batteries and buttons (6.55% each), large seeds and alimentary boluses (4.91% each), glass, marbles, toothpicks, magnets, unidentified plastic objects (toy parts) (3.27% each), and needles, screws, nails, keys, hair pins, shattered glass, pencils, and plastic lenses (1.64% each) (Table 3).

**Figure 1:**
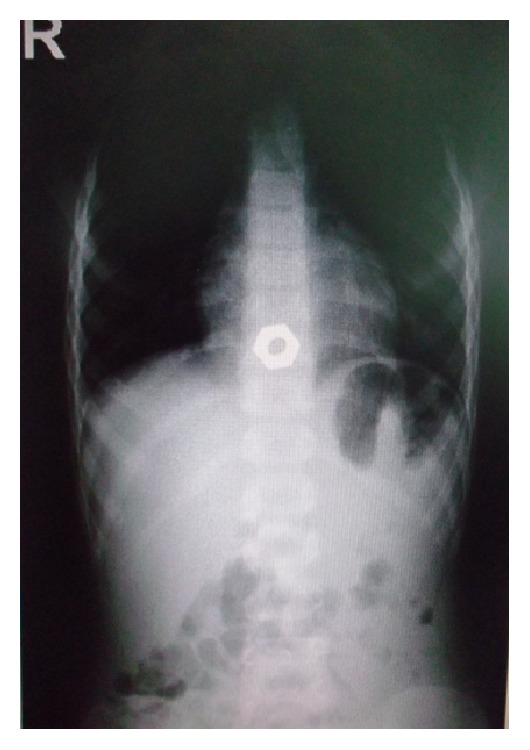
Foreign body at the cardia of a 6-year-old boy.

Routine X-ray found the foreign body only in 42 of the cases; the 19 patients with negative X-ray ingested radio-transparent materials (Figure 1).

An esophagogastroduodenoscopy was performed within 24–72 hours from ingestion due to late hospital presentation and lack of an emergency endoscopy service that affected weekend hospital presentations; some patients presented after food ingestion, which also delayed the endoscopy. Endoscopy was performed also in the 19 patients in which the foreign body could not be identified using X-ray (the anamnestic data indicated the ingestion of a radio-transparent object or the clinical symptoms were important). In 25 cases (40.98%) the endoscopy did not help to identify the foreign body and in 36 children we were able to find the ingested material. We divided the patients into three categories, as follows: negative X-ray with endoscopic findings (foreign bodies or mucosal injuries, such as erosions or superficial ulcerations), positive X-ray with endoscopic findings, and positive X-ray with negative endoscopy (neither foreign bodies nor mucosal injuries; all these patients had ingested blunt metal objects). The correlations between X-ray and endoscopy are described in Table 4.

**Figure 2:**
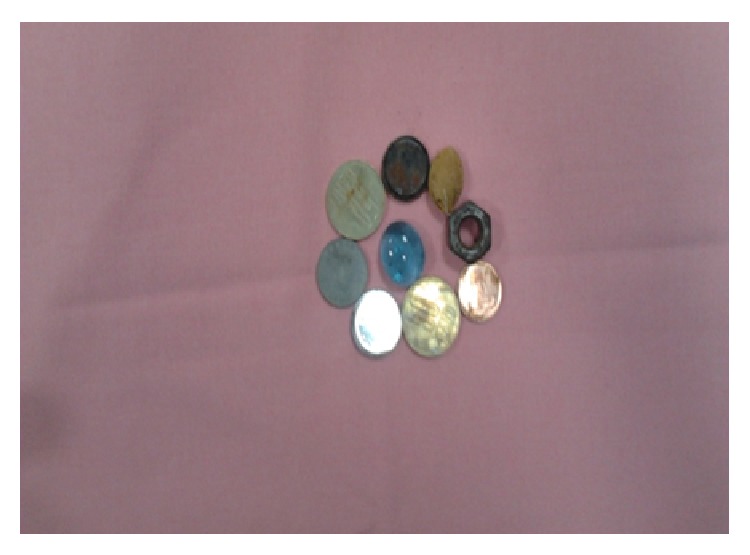
Foreign bodies retrieved in the patient series.

Of the 36 children with positive endoscopy, 19 objects (31.14%) were removed using a polypectomy snare, while extraction failed in 17 patients (27.86%) (Figure 2).

In 59 of the patients endoscopic examinations did not reveal any underlying condition such as eosinophilic or reflux esophagitis or other associated pathologies such as esophageal diverticula; two of the children were previously diagnosed with caustic esophageal stenosis.

28 foreign bodies were passed naturally within 3 to 20 days; in 14 cases they were never found, despite parental surveillance, so we presumed they passed unnoticed. In one case with late presentation a battery was stuck in the esophageal folds. The battery was removed in the otorhinolaryngology service, but a week later the patient returned with acute respiratory distress; despite intensive treatment, a prolonged and unfavorable evolution linked to the anamnestic data required an upper digestive endoscopy that revealed a tracheal-esophageal fistula. Subsequent complications included esophageal stenosis treated by endoscopic bougienage.

## 4. Discussions

Foreign body ingestions are a public health issue due to their high frequency, especially in children and older patients. The majority of ingested foreign bodies pass spontaneously through the gastrointestinal tract without causing injury; however, according to Louie and Bradin, 10–20% will require nonsurgical intervention and less than 1% will require surgery [6]. In our study we found a median age of 3.25 ± 4.7 years, with more than half of the patients (55.73%) being under 6 years of age. Gender distribution showed a slight male predominance. These findings are consistent with other reports such as the ones of Adhikari and other authors regarding age and gender distribution [7, 8]. Discussions indicate that the high prevalence of foreign body ingestions in younger children is due to the exploratory habits of these children and that gender involvement is not significant in this pathology. As far as the clinical presentations are concerned, we had a large proportion of patients presenting with abdominal pain (55.73%), followed by vomiting in 34.42%, as well as 29.50% of asymptomatic children. These results are different from reports of working groups and authors like Arms, Abbas, and Conners that found asymptomatic children in variable percentages of 25%, 50%, and 55%, respectively [8–11]. In our opinion, these differences could be attributed to the nature of the ingested objects. In our series we performed a multiple regression analysis which indicates that the clinical symptoms are significantly influenced by the shape of foreign body (pointy-shaped objects being associated with clinical symptoms) (*r* partial = 0.56, *p* < 0.01), followed by the time between the event and the presentation (symptoms occurring in patients who presented within the first hours from ingestion) (*r* partial = 0.45, *p* < 0.01), while the last predictive factor is the age of the patient, less than 5 years (*r* partial = 0.34, *p* < 0.000557) (Table 2). Other authors found a significant association between location, size, and time elapsed from the accidental ingestion [12]. Although we expected a significant correlation between the size of the foreign body and the clinical features, this could not be objectified probably due to the nonhomogenous distribution of ages among our patient series (teenagers ingesting small foreign bodies had less clinical symptoms than toddlers ingesting small pieces). Our study included a great variety of swallowed objects, ranging from coins to magnets and unidentified plastic objects. We found that the most frequently ingested objects were coins, which were reported in 26.23% of the patients, with similar results being reported by Rybojad et al. [12]. Smaller coins passed without any incident while larger coins were associated with vomiting and abdominal pain in 7 cases. A number of 4 children (6.55%) had ingested alkaline disk batteries from toys, remote controls, and watches. We noticed that the ingestion of disk batteries and plastic toys had a higher incidence in children of younger ages; this might be explained by age-specific preferences for different kinds of toys: acoustic and moving toys in children under the age of 3 and building games in older ones. The plain X-ray is an important diagnostic tool, being the main initial imaging work-up. In our series, the foreign body was found using this method only in 42 of the cases. In the rest of the cases, the patients had either swallowed a nonradiopaque object or eliminated it prior to admission and passed unnoticed. In our study, the X-ray identification rate was 68.85%, which is similar to rates reported by working groups' studies and the research of Litovitz et al. and Shastri et al. that ranged from 64% to 96.04% [8, 13, 14]. We agree with other authors who stated that the optimal modality for removing the foreign body is largely dependent on many factors, including the patient's age, the clinical condition, the size, shape, and type of the foreign body, the anatomic location, the technical possibilities, and the skills of the endoscopist [15]. Upper digestive endoscopy is the most common method used to retrieve ingested foreign bodies, as discussed by Waltzman et al. [16]. 31.14% of patients in this study were managed via successful endoscopic removal; this rate is higher compared to that in Yang's study (23%) and lower compared to that in Pokharel et al.'s study (98.06%) [17, 18]. These differences could be due to the variable time elapsed until hospital presentation, the size and type of objects swallowed, or the different technical resources in pediatric centers. Only 2 of the patients that underwent endoscopic removal presented complications such as minor and self-limited bleeding. In our series of children presenting with foreign body ingestions we did not find any underlying condition of the esophagus, except for two children previously diagnosed with esophageal stenosis. As Kramer et al. reported, eosinophilic esophagitis, esophageal stenosis, or diverticula may favor esophageal impaction [19]. We only had one case of unfavorable evolution in a 2-year-old girl with a disk battery impacted in the upper cervical esophagus and extracted in the otorhinolaryngology service; the patient presented afterwards with complications including tracheal-esophageal fistula and bronchopneumonia and subsequently with high esophageal stenosis that required endoscopic dilation. No deaths were recorded, which is consistent with the low mortality rates associated with foreign body ingestions worldwide reported by other studies [1]. However, in our series we had a high proportion of failed endoscopic removals, which were almost similar to the successful attempts (27.86% versus 31.14%). This is due to our limited resources in terms of endoscopic retrieval devices, which are frequently inappropriate for the respective objects. All the foreign bodies that could not be retrieved via endoscopy were eliminated spontaneously within 3 to 20 days, depending on child's intestinal habits. Aside from these, another 11 objects that were not found during the initial endoscopic examination were later found by parents in the children's feces, thus adding up to a total of 28 eliminated foreign bodies (45.90%). Despite careful surveillance reported by parents, 14 objects (22.95%) were never found. Analyzing this data, our study reports rather negative results, since the proportion of removals is lower than the proportion of spontaneous eliminations. Late presentation along with the lack of early endoscopy plays an important part in subsequent complications and in the inability to find the foreign objects; in approximately half of the cases where the foreign body was not found, upper digestive endoscopy detected erosive gastric mucosal lesions, which indicated that the object had passed. The large majority of patients (60 children, 98.36%) were dismissed in good condition; all the parents received instructions on possible alarm symptoms indicating an obstruction or perforation, as well as recommendations for daily examination of their children's stools. We agree with Cheng and Tam's recommendation for parents to search their children's feces for the foreign bodies on a daily basis [20]. Medical therapy for objects that cannot be reached with endoscopic devices depends on the presentation type and complications; prokinetic agents and laxatives may be an alternate approach, with a 100% success rate before any surgical intervention [6]. Early recognition of foreign body ingestions and appropriate management can significantly reduce morbidity due to complications. We agree with Palta et al.'s report that an increased awareness of the parents and people involved in the institutional care settings (nurseries, kindergartens, centers for children with neuromotor disabilities, and child psychiatry services) along with an active surveillance during daily activities is essential in order to establish protective rules that help to keep hazardous materials out of the children's reach [21]. At the same time, in children under the age of 3 the avoidance of toys containing small parts that can be easily ingested or inhaled is the most effective prevention method, yet this recommendation is frequently ignored by the parents. In our opinion, the education of parents and health professionals working with children might be an effective prevention method. Batteries and sharp objects may lead to severe complications and the preschool-age children are at a high risk for such events [16]. The limitations of this study are mainly that this was a retrospective-descriptive review providing data from a single pediatric gastroenterology center; further research is necessary.

## 5. Conclusions

Foreign body ingestions in children appear more frequent at younger ages and in the absence of associated conditions of the esophagus. We found a relatively low frequency of this pathology. Clinical findings depend on the shape of the ingested object, time of referral, and patient's age; some children may be completely asymptomatic. The rate of severe complications is low. We report a large proportion of foreign bodies that could not be identified and/or removed. In our opinion, the diagnosis and management of this pathology in limited endoscopic settings are still difficult due to the absence of standardized guidelines and poor technical resources; emergency endoscopic services in pediatric tertiary care centers are mandatory.

## Figures and Tables

**Table 1:** Clinical symptoms in the patient series.

Clinical presentation	Number of patients	%
Asymptomatic	18	29.50
Abdominal pain	34	55.73
Vomiting	21	34.42
Foreign body sensation	7	11.47
Hematemesis	2	3.27
Drooling and food refusal	1	1.64
Stridor and cough	1	1.64

**(a):** 

Multiple correlation	Estimated value
Multiple correlation coefficient *r*	0.92344
Multiple *r* ^2^	0.85274
*F*(6, 1161)	20.68154
*p*	0.00000
Std. err. of estimate	0.32562

**(b):** 

Partial correlation	Correlation interval (beta)	Std. err.	*T*	*p* 95% confidence interval
Intercept			−6.54998	0.000000
Patient factors				
Age	0.348487	0.070137	3.54287	0.000557
Sex	0.093035	0.065924	1.41125	0.160654
Institutionalization	−0.022719	0.065342	−0.34769	0.728660
Foreign body factors				
Size	−0.102692	0.066169	−1.55198	0.123196
Material	−0.010585	0.067656	−0.15646	0.875925
Shape	0.565067	0.066849	6.95704	0.000000
Time to presentation	0.450815	0.062466	5.61612	0.000000

**Table 3:** Types of foreign bodies encountered in the patient series.

Foreign body	Number of patients	%
Coins	16	26.23
Other metal objects	8	13.11
Bones	5	8.19
Batteries	4	6.55
Buttons	4	6.55
Large seeds	3	4.91
Alimentary boluses	3	4.91
Glass, marbles, toothpicks, magnets, and unidentified plastic objects (toy parts)	2 each	3.27 each
Needles, screws, nails, keys, hair pins, pencils, plastic lenses, and shattered glass	1 each	1.64 each

**Table 4:** Correlations between X-ray and endoscopic findings.

		Cases with positive X-ray		
		42		

Negative X-ray and endoscopic findings		Positive X-ray and endoscopic findings		Positive X-ray and negative endoscopy
Mucosal injuries	Foreign bodies	Foreign bodies	Mucosal injuries	12
5	14	22	8	

	36			
	Cases with foreign bodies identified using endoscopy			
